# Protein Fitness Prediction Is Impacted by the Interplay of Language Models, Ensemble Learning, and Sampling Methods

**DOI:** 10.3390/pharmaceutics15051337

**Published:** 2023-04-25

**Authors:** Mehrsa Mardikoraem, Daniel Woldring

**Affiliations:** 1 Department of Chemical Engineering and Materials Science, Michigan State University, East Lansing, MI 48824, USA; 2 Institute for Quantitative Health Science and Engineering, Michigan State University, East Lansing, MI 48824, USA

**Keywords:** machine learning, protein fitness prediction, embeddings, sequence representation, imbalanced assay-labeled datasets, sampling methods, ensemble learning, MCDA, TOPSIS

## Abstract

Advances in machine learning (ML) and the availability of protein sequences via high-throughput sequencing techniques have transformed the ability to design novel diagnostic and therapeutic proteins. ML allows protein engineers to capture complex trends hidden within protein sequences that would otherwise be difficult to identify in the context of the immense and rugged protein fitness landscape. Despite this potential, there persists a need for guidance during the training and evaluation of ML methods over sequencing data. Two key challenges for training discriminative models and evaluating their performance include handling severely imbalanced datasets (e.g., few high-fitness proteins among an abundance of non-functional proteins) and selecting appropriate protein sequence representations (numerical encodings). Here, we present a framework for applying ML over assay-labeled datasets to elucidate the capacity of sampling techniques and protein encoding methods to improve binding affinity and thermal stability prediction tasks. For protein sequence representations, we incorporate two widely used methods (One-Hot encoding and physiochemical encoding) and two language-based methods (next-token prediction, UniRep; masked-token prediction, ESM). Elaboration on performance is provided over protein fitness, protein size, and sampling techniques. In addition, an ensemble of protein representation methods is generated to discover the contribution of distinct representations and improve the final prediction score. We then implement multiple criteria decision analysis (MCDA; TOPSIS with entropy weighting), using multiple metrics well-suited for imbalanced data, to ensure statistical rigor in ranking our methods. Within the context of these datasets, the synthetic minority oversampling technique (SMOTE) outperformed undersampling while encoding sequences with One-Hot, UniRep, and ESM representations. Moreover, ensemble learning increased the predictive performance of the affinity-based dataset by 4% compared to the best single-encoding candidate (F1-score = 97%), while ESM alone was rigorous enough in stability prediction (F1-score = 92%).

## 1. Introduction

Proteins are biological machines involved in almost all biological processes [1,2,3,4]. These molecules are made of amino acids that fold into 3-dimensional structures and perform life-sustaining biological functions [5]. Protein engineering practices aim to modify proteins to redirect what has already evolved in nature and address the industrial and medical needs of modern society [6,7]. This has been a challenging task due to the astronomical number of possible mutations and the complex sequence–function relationship of the proteins (i.e., fitness landscape) [8]. Protein fitness—a measure for how well a protein performs a task of interest—is influenced by a variety of factors, including its structure, stability, and interactions with other molecules. In protein engineering campaigns, where protein function is modified by experimental approaches rather than natural selection, proteins with high fitness are those that perform well within relevant experimental assays, whereas proteins with low fitness result in reduced activity, altered specificity, or decreased stability. To overcome the challenge of finding high-fitness proteins among mostly non-functional mutants, various experimental and computational techniques were developed. Recently, machine learning (ML) has shown promise as a tool to supplement already established techniques, such as rational design and directed evolution [9,10,11,12]. Unlike directed evolution, ML models can learn from non-functional mutants instead of simply discarding them during enrichment for functional clones. ML-assisted protein engineering, therefore, has potential as a time-efficient and cost-effective approach to searching for desired protein functionality. This provides a unique opportunity to create smart protein libraries, elevate and accelerate directed evolution and rational design strategies, and finally, enhance the probability of finding unexplored high-fitness variants in the protein fitness landscape [13,14,15]. Machine learning methods have attained a high success rate in predicting essential protein properties (i.e., protein fitness) including secondary structure, solubility, binding affinity, flexibility, and specificity [16,17,18,19,20]. Despite these recent milestones, in order to obtain generalizability and robustness in ML models, further explorations in different protein fitness prediction tasks and training details are required.

Dealing with protein fitness landscape challenges will require us to view proteins from a new perspective that supplements our biochemical knowledge with lessons from written languages. Recent advances in ML and artificial intelligence have applied natural language processing (NLP) methods to identify context-specific patterns from written or spoken text. NLP tasks learn how words function grammatically (syntax) and how they deliver meaning within themselves and in surrounding words (semantics) [21,22]. This has given rise to virtual assistants with voice recognition and sentiment analysis of text from diverse languages [23,24]. Similarly, protein engineering can leverage these NLP tools—treating a string of amino acids as if they were letters on a page—to understand the language of proteins, providing a promising route to capture nuances (e.g., epistatic relationships, functional motifs) in complex sequence–function mappings [25,26]. The rapid expansion of publicly available protein sequence data (e.g., Uniprot [27], SRA [28]) further supports the use of big data and language models in the domain of protein engineering [29]. Self-supervised language models learn the context of the provided text by reconstructing the masked tokens/linguistic units of the text string using the unmasked parts. For the context of protein engineering, pre-trained protein language models—carrying valuable information about the epistasis/interaction of amino acids—can be applied to downstream tasks by extracting the optimized weight functions as a fixed-size vector (embedding) [25,30,31]. Among early embedding developments, Alley et al. introduced UniRep [32], a deep learning model that was trained on 24 million unique protein sequences to perform the next amino acid prediction tasks for extracting information about the global fitness landscape of proteins. Rives et al. trained ESM, a language model for masked amino acid prediction tasks, on over 250 million protein sequences [33]. The learned representations—including UniRep [32], ESM [33], TAPE [34], and ProteinBERT [35]—have generated promising results in diverse areas such as predicting protein fitness, protein localization, protein–protein interaction, and disease risk of mutations in terms of improved prediction scores, increased generalizability, and mediated data requirements [36,37,38,39,40]. Using embeddings for sequence representations (transfer learning) enables knowledge transfer between protein domains and future prediction tasks by further optimizing the already-learned weights. For example, Min et al. obtained a 20% increase in the F1-score (the harmonic mean of precision and recall) for a heat shock protein identification task when training their NLP-based model, DeepHSP [41], on top of pre-trained representations.

In this study, we perform protein sequence fitness prediction with ML techniques to demonstrate how model performance varies given the choice of protein representation, protein size, and the biological attribute (e.g., binding affinity and thermal stability) to be predicted. This work provides actionable insights for effectively building discriminative models and improving their prediction scores via sampling techniques and ensemble learning. As efficient use of embedding methods on experimental datasets is in its infancy, rigorous studies are needed to gain new insights into the performance of the pre-trained models given various training conditions and distinct biological function predictions. Importantly, embedding methods have been trained over millions of protein sequences in public databases and have produced high performance in certain fitness tasks (e.g., stability prediction), while they may not do as well in all fitness prediction tasks. To this end, we used two large datasets that were representative of common protein engineering tasks. First, we leveraged a highly imbalanced dataset (93% non-functional; Table 1), consisting of our previously described affinity-evolved affibody sequences [42] to explore NLP-driven practices. We then expanded our analysis to include thousands of protein sequences labeled with their experimentally measured stabilities (melting temperatures, Tm) obtained from the Novozymes Enzyme Stability Prediction (NESP) dataset [43]. Thus, with our two datasets having unique attributes, we were well positioned to address multiple questions: (i) How do different representation methods perform in predicting distinct fitness attributes such as stability or affinity? (ii) How do sampling methods perform in the imbalanced protein datasets? (iii) Is ensemble learning over different protein representations helpful in boosting the performance of discriminative models? (iv) How do we rank model performances while using multiple conflicting metrics in ML prediction tasks? By addressing these challenges, we also gain direct insights for model interpretation and reveal the features that are most important for discriminating between fit and non-fit sequences (Figures S1, S3, and S4). We discovered that oversampling (especially SMOTE) generally outperformed the undersampling techniques In addition, ensemble over representations greatly improved the predictive performance in the affibody data both using single and multiple performance metrics via multiple criteria decision analysis (MCDA) [44]. For protein representations (e.g., single encoders), UniRep and One-Hot outperformed other methods in the affibody (affinity) dataset while ESM achieved the best score in stability prediction in NESP. Finally, it was observed that the performance of various protein representation methods is strongly impacted by protein sequence length.

## 2. Materials and Methods

### 2.1. Obtaining Experimentally Labeled Sequence Data

Two different datasets with varying data characteristics were explored. The first is our experimental data of affibody sequences that previously were iteratively evolved for binding affinity and specificity against a panel of diverse targets [42]. The second collection of labeled protein sequences was obtained from the recently released Kaggle dataset wherein numerous proteins (*n*= 18,190) of various lengths are labeled according to their thermal stability (Tm). This dataset, NESP, was filtered to only include sequences characterized at pH = 7. For the affibody dataset, raw sequence data were cleaned by removing any sequences that contained stop codons or invalid characters. Afterwards, the frequency of each unique sequence in the experimental steps was tabulated. Infrequent sequences appearing fewer than ten and four times (within magnetic activated cell sorting (MACS) and fluorescent activated cell sorting (FACS), respectively) were treated as background and removed from the analysis. Note that the more stringent frequency removal for MACS was mainly due to the experiment type and higher probability to introduce noise in the dataset. After removing the background, sequences from MACS and FACS were combined to form the final high-fitness population of binders. The non-binding population included the initial affibody sequence pool, which did not appear in the enriched population of the binder sequences. The initial affibody sequences that were within one hamming distance (i.e., a single amino acid mutation) of any enriched sequence were removed as well to account for potential errors encountered during deep sequencing. All affibody sequences were exactly 58 amino acids in length with mutations present at up to 17 of these positions.

### 2.2. Obtaining the Sequence Representations

We obtained four different numerical representations for our sequence data: One-Hot and physiochemical encoding, UniRep, and ESM embeddings. One-Hot encoding refers to building a matrix (amino acids × protein length) and filling it with one when there is a specific amino acid in the given position, filling the rest with zeros. For physiochemical encoding, we used the modlamp [45] package in python, which is used for extracting the physical features from protein sequences. There were two types of physical features represented in the modlamp package (global and peptide descriptors). All the global (e.g., sequence length, molecular weight, aliphatic index, etc.) and local physiochemical features based on the Eisenberg scale were extracted for this analysis (twenty in total).

Embedding refers to continuous representation of the protein sequence in a fixed-size vector, and it should contain meaningful information about proteins [46]. For example, in the embedding visualization of amino acids in low dimensions for both UniRep and ESM, similar amino acids (in terms of size, charge, hydrophobicity, etc.) were close to each other. For UniRep representation, we used the 1900 dimension and mean representation over layers. We used Jax_UniRep for obtaining the UniRep embeddings, https://github.com/ElArkk/jax-unirep (accessed on 20 August 2022). UniRep uses the mLSTM structure for performing next-token prediction, and it was trained on 24 million sequences in the Uniref50 dataset with 18 M parameters. For ESM, we chose ESM2 [47] with 1280 vector dimensions and 650 M parameters and means over layer representations. GitHub for ESM is https://github.com/facebookresearch/esm (accessed on 21 January 2023).

### 2.3. Sampling and Splitting

Sampling refers to choosing a random subset of data to represent the underlying population. Three different sampling methods were tested for our severely imbalanced affibody dataset: undersampling, random oversampling, and the synthetic minority oversampling technique (SMOTE) [48]. Due to the sparse and rugged nature of the protein fitness landscape, it is common for experimental data obtained in the protein domain to be highly imbalanced. One practical approach for resolving the imbalanced dataset issue is using sampling techniques when training the dataset. Oversampling is randomly repeating the minority class examples; thus, it could be prone to overfitting in comparison to undersampling. However, undersampling may discard useful information, especially in severely imbalanced datasets, as it is removing many samples from the majority class. SMOTE is a more recent addition to sampling methods, and it is oversampling the minor population by synthetically generating more instances that are highly similar to the minority class. While SMOTE has shown promising results in increasing the prediction performance for various imbalanced datasets [49,50,51], there are also studies indicating undersampling superior performance compared to oversampling methods [52,53]. As a result, we examined the performance of all three sampling techniques to validate which sampling method performs well within our wet-lab protein dataset over different encoding methods.

For splitting the datapoints within the test set in an imbalanced dataset, sampling equally from each class may lead to an overestimation of the model performance [54]. As a result, we made sure that the test set distribution follows the initial data distribution (93% naïve vs. 7% enriched).

### 2.4. Algorithm Selection and Training Details

For classification, logistic regression (LR) was chosen and L2 penalization (Ridge) was used to reduce the likelihood of overfitting. We reasoned that a simple logistic regression enables a fair comparison between cases. One regression task was also implemented over the NESP dataset with random forest regressor (RFR). We used regression to observe how models perform with increasing the prediction challenge, from binary prediction to actual label prediction. The rationale for using RFR was that linear regression model was not viable to meet the prediction task complexity. For a fair comparison between protein encoding performances in regression, the RFR hyperparameters, max number of estimators and max_depth, were optimized with OPTUNA [55].

### 2.5. Ensemble Learning

To improve the predictive performance of protein encoding predictions, we developed a framework that combines various encoding methods. We experimented with two approaches: **concatenation and voting**. In concatenation, the encodings were combined by adding them together, and we used the resulting representation as input for our predictive model. In voting, separate predictive models for each encoding method were trained. The final prediction was then calculated with the majority-voted label over a fixed test set.

### 2.6. Metrics and Statistical Analysis

**Figure 1:**
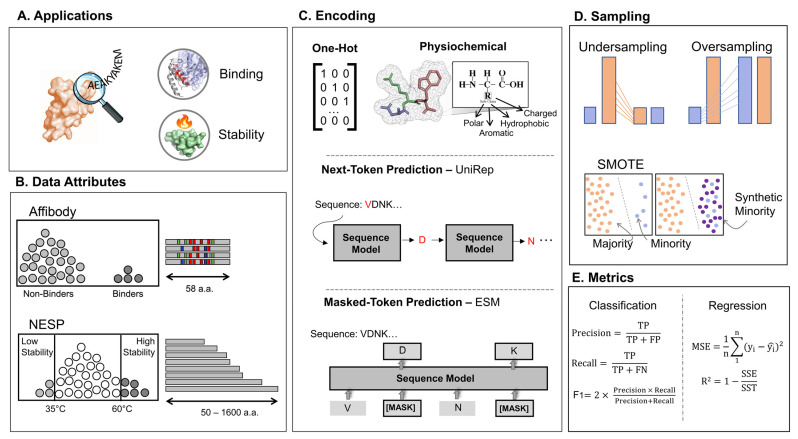
Overview of the implemented techniques, data attributes, and evaluation metrics. (**A**) Illustrates the use of sequence–function mapping to identify protein sequence functionality (e.g., therapeutics, diagnostics, enzymatic function). (**B**) Data attributes for the two datasets used in this study. The first dataset includes high-fitness protein binders among a pool of non-binder affibody sequences with up to 17 mutation sites. The other dataset includes a wide array of proteins with their associated melting point. (**C**) One-Hot encoding, physicochemical encoding, and pre-trained models were used to encode the protein sequences present in our datasets. All present protein amino acid information is in a machine-readable format, but in different ways. One-Hot encoding converts each amino acid to a binary vector of all 0 s but 1 where it belongs to its position in the matrix. In physicochemical encoding, each amino acid is represented by its physiochemical characteristics, such as polarity, charge, size, etc. Pretrained models are trained over a large corpus of unlabeled data capturing the syntax and semantics of protein language via NLP-driven models, such as next-token prediction (e.g., UniRep) and masked token prediction (e.g., ESM). (**D**) The sampling methods used in this study are undersampling, oversampling, and synthetic minority oversampling techniques (SMOTE). (**E**) The main metrics used for evaluating the performance of prediction tasks (classification and regression) are defined (a complete list of performance metrics are listed in Figure S7).

One key metric we used for analyzing classification performance is the F1-score. By considering both precision and recall (Figure 1E), the F1-score is particularly well suited for evaluating the highly imbalanced data within our study (Table 1). Therefore, the model is trained to identify the positive instances among all positive predictions and minimize missing out the positive instances while predicting classes. Note that other classification metrics, such as confusion matrix values (TP, TN, FP, FN), are reported in the supplement figures. For regression analysis among NESP data, we used mean squared error (MSE) and R^2^ to indicate how the models perform. MSE is the mean of the square of differences between the actual labels and the predicted values in the test set while R^2^ represents the variation explained by the independent variables.

Experiments were implemented with multiple random seeds (20 in affibody and 30 in NESP dataset) to obtain a distribution of performances for each pair of encoding and sampling methods in each fitness prediction task. Then we implemented multiple statistical tests to confirm if the obtained differences were significant. Analysis of variance among the groups was performed with ANOVA [56]. After obtaining significant results in ANOVA, post hoc methods were implemented to account for family-wise error rates. Here, we implemented two post hoc methods, Bonferroni [57] and Tukey [58], for adjusting the *p*-values and reducing the risk of type-1 error. The null hypothesis assumes that the performances of the methods are similar and when rejected, we consider the methods to be statistically significant in their obtained output. The results for multiple seeds are shown with violin plots where the white dots represent the mean values. A complete collection of statistical analyses for comparing the significance among the means are located in the supplementary information.

### 2.7. Multiple Criteria Decision Analysis (MCDA)

We used F1-score as our primary classification metric in classification to optimize the algorithms based on finding the rare positive sequences. Depending on specific applications, the user may need to choose different criteria for analyzing the ML predictive performance. Note that it is also generally advised to use multiple metrics to establish more rigorous analyses, specifically in imbalanced datasets [59,60]. Therefore, we incorporated five more classification metrics in addition to F1-score and implemented MCDA, which is a robust approach for decision making (i.e., ranking alternatives based on multiple, often conflicting, criteria). In our study, the alternatives are the choice of protein representation within different sampling methods. The criteria (classification metrics) used for this MCDA include F1-score, false positive rate (FPR), true positive rate (TPR), precision, negative predictive value (NPV), and false discovery rate (FDR). FPR and TPR measure the model’s ability to identify the positive and negative classes. Precision quantifies the number of correctly positive classes among all being predicted as positive, while NPV is measuring this for the negative class. FDR measures the number of false positives over all instances that are predicted as positives.

The performance of each encoding and sampling technique was recorded based on all six mentioned criteria. For implementing the decision-making, we chose a well-established and widely used MCDA method: the technique for order of preference by similarity to ideal solution (TOPSIS) [61]. TOPSIS finds the optimal solution rooted in the idea that the best alternative should have the minimum Euclidean distance from the positive ideal solution and maximum distance from the negative ideal solution.

For implementing TOPSIS, the PyTopsis package in python was used, https://github.com/shivambehl/PyTopsis (accessed on 4 April 2023). It requires three inputs: the decision matrix (i.e., alternative scores within chosen criteria), list of weights (i.e., criteria importance), and list of signs (−1 indicates the criteria should be minimized while 1 implies maximizing). Following this structure, we built our decision matrix and assigned directions that each criterion should be optimized in classification. Assigning weights to criteria can be implemented either by the decision-maker’s opinion (subjective weighting) or a numerical process over the decision matrix (objective weighting). We implemented both methods and compared their results in ranking the alternatives. For subjective weighting, we assigned a slightly higher weight for precision and FPR metrics to prioritize identifying the positive instances. For objective ranking, the Shannon’s entropy method [62] was implemented to measure the entropy based on the given decision matrix. The formula used to calculate weights based on the entropy are in the following where *x_ij_* is each entity in the matrix, *n* is the number of alternatives, and m is the number of criteria.

Normalizing the decision matrix value

(1) $$r_{ij}=\frac{x_{ij}}{\sum_{i=1}^{n}x_{ij}}$$

Calculating Entropy for each criterion

(2) $$E_{j}=-k\sum_{i=1}^{n}r_{ij}\ln r_{ij},\;\;\;k=\frac{1}{ln(n)}$$

Calculating weight for each criterion

(3) $$w_{j}=\frac{1-E_{j}}{\sum_{j=1}^{m}\left(1-E_{j}\right)}$$

The results from TOPSIS need to be validated via statistical methods to ensure the correct ranking among alternatives (i.e., difference between performances is not random but significant). Therefore, we applied multivariate analysis of variance (MANOVA) [63] followed by a post-hoc method, Tukey [58], to analyze the result significance overall and between pair of alternatives, respectively.

Figure 1 provides an overview of the data attributes (e.g., protein size, protein fitness) that will be predicted and alternatives (e.g., protein encodings within different sampling methods) that will be compared.

## 3. Results

### 3.1. Sequence-Function Mapping Obtained from High-Throughput Selection Methods and Deep Sequencing Affibody Dataset

To investigate the impact of feature representation, ensemble learning, and sampling methods, several prediction tasks were leveraged. We performed a classification task on the obtained sequences to predict the scarce high affinity binder class among the pool of non-binder class in the affibody data. For NESP dataset, in the classification task, we simplified the data by choosing two classes of low- (Tm ≤ 35 °C) and high-stability (Tm ≥ 60 °C). In addition, regression was implemented to increase the prediction difficulty and to observe how protein encodings perform relatively. The models were tasked with predicting the stability (Tm) value, and all the sequences with measured pH = 7 were included. The details of obtained sequences after cleaning and the type of prediction tasks are reported in Table 1. **Note that the NESP results will be overviewed in** Section 3.5 **and supplementary information.**

**Table 1:** Dataset attributes and prediction tasks.

Dataset	Task	Fitness	Model	Attributes
Affibody	Classification	Binding Affinity	Logistic Regression	82,663 non-binders
				6077 binders
NESP	Classification	Stability	Logistic Regression	3743 high-stability
				1311 low-stability
NESP	Regression	Stability	Random Forest Regressor	18,190 total

### 3.2. Physiochemical Feature Encoding, Interpretable Yet Lower Predictive Capacity

**Figure 2:**
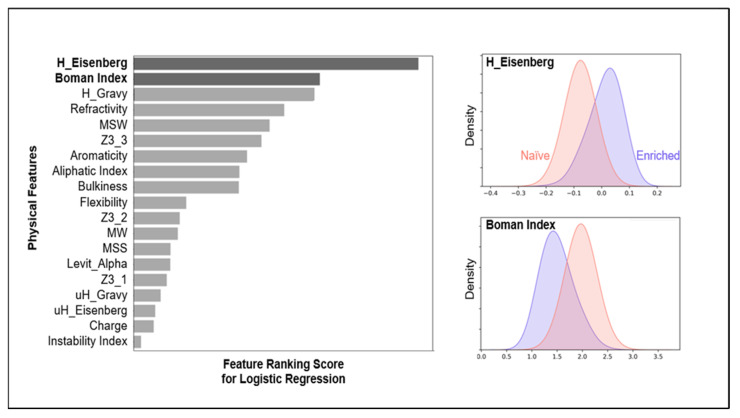
The lead physical features in naïve and enriched class discriminations in affinity-based data were H_Eisenberg, Boman Index, and H_Gravy. Gravy and Eisenberg capture hydrophobicity scales. The Boman Index is a measure of the protein’s ability to interact with its environment based on the solubility of individual residues. The enriched proteins in our library have gone through negative screening and are specific to their target. Therefore, there is a shift to a lower Boman index for this population. Note that the plot is the result of oversampling, SMOTE, in the logistic regression task.

**Figure 3:**
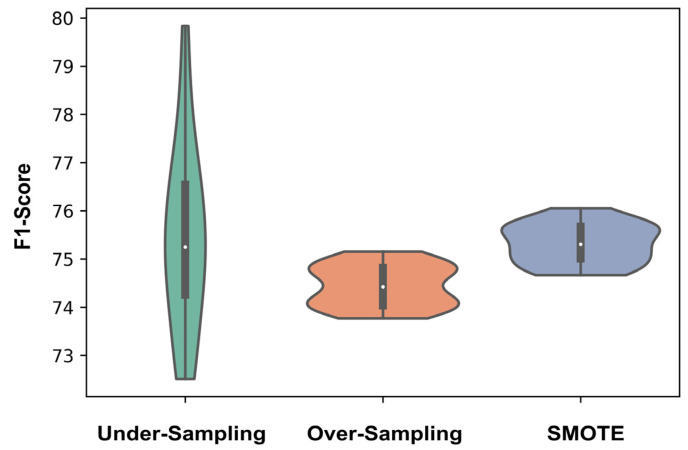
When physical features were used to encode the affibody sequences, the mean F1-score was 75.5% with SMOTE. Both SMOTE and undersampling methods were similarly effective, with no significantally signficant difference in performance (i.e., did not reject the null hypothesis). The violin plots are created over 20 random seeds for each sampling method.

The classification results in physiochemical encodings are shown in Figure 2 and Figure 3. We ranked the leading features in discriminating non-binder and binder classes and listed the encoding method’s F1-score in different sampling methods. The physiochemical encoding performance was not among the lead encoding methods, yet it achieved a high F1-score with only 20 features. It also provided insights on how physical features correlate with each other in the given data (Figure S1).

### 3.3. Comparison over All the Encoding and Sampling Methods

**Figure 4:**
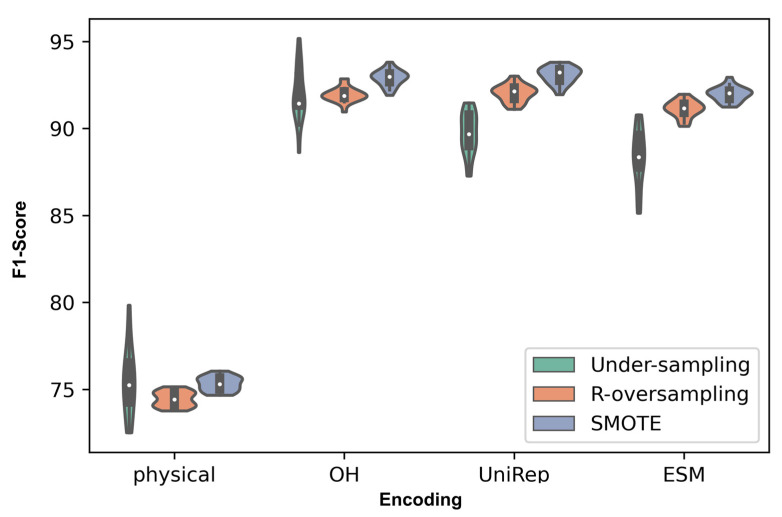
Performance analysis of encoding methods highlights the shortcomings of physical features and strength of the SMOTE sampling method. Protein sequences encoded using physical features, One-Hot, UniRep, and ESM were used to perform classification tasks among the affibody dataset. Within each encoding method, undersampling, random oversampling, and SMOTE sampling methods were evaluated. The resulting F1 scores over 20 random seeds are shown here as violin plots. The obtained *p*-value from ANOVA was 9.52E−190, which indicated a significant effect among comparisons. Post-hoc results for ranking methods are shown in Table S1, which consolidates the mentioned conclusions in the caption.

Once the lead physical features for high-affinity binders were determined, we demonstrated the performance of different protein representations within our selected sampling techniques. The prediction performance indicates that each encoding method performed differently in predicting the fitness of proteins, and One-Hot and UniRep were the top performers. In addition, among the samplings, SMOTE boosted the F1-score in almost all cases. Figure 4 exhibits the F1-score distributions within 20 different random seeds.

### 3.4. Increased Generalizability and Predictive Performance via Ensemble Learning

**Figure 5:**
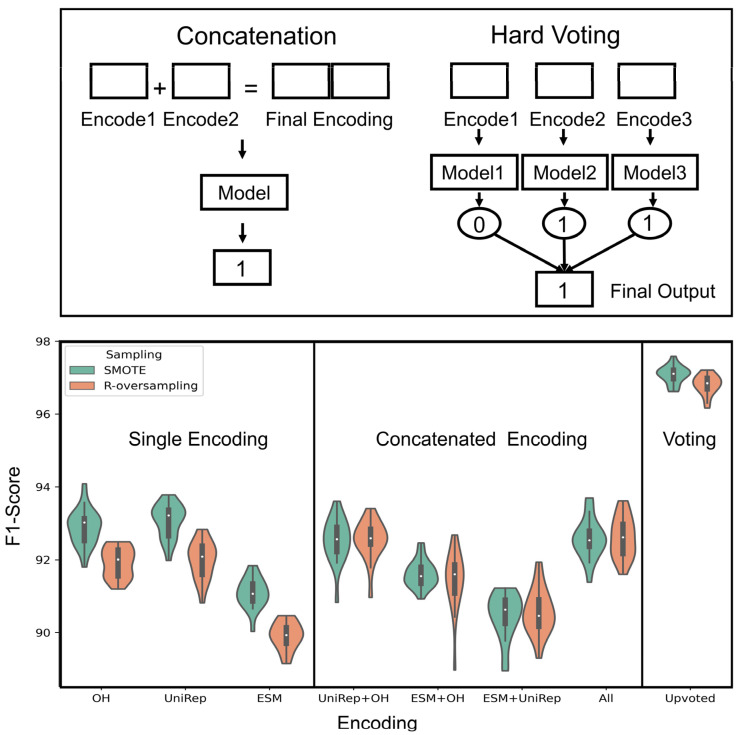
Voting substantially improved the predictive performance in all random initializations over different encoding methods. The plot above has three regions from left, respectively; it includes single encoding methods, concatenation of encodings, and voting of predictions. The vote was performed such that each encoding went through a predictive model over the same dataset. Then, the final prediction was obtained by majority voting. It is insightful how voting increases the models’ robustness and generalizability. The concatenation performed similarly or worse than the best model in single encodings. The best model among all predictions was Upvote with oversampling methods with Mean-F1-score = 97% and Mean-F1-score = 96.80% (no statistical significance among oversampling performances in upvoting). Refer to the supplementary material for a summary of the statistical analysis and confusion matrix plots (Table S2, Figure S2).

Due to the varying performances of the protein encodings, we postulated that ensemble learning increases the models’ predictive performance. As oversampling performed better than undersampling in three out of four encoding methods, we exclusively analyzed the ensemble learning for the two oversampling types (i.e., R-oversampling, and SMOTE). The physical encoding for this analysis was discarded since its performance was not as potent as the other encodings. Figure 5 represents the ensemble technique, voting, which remarkably enhanced the performance with respect to all the methods with a mean F1-score = 97% over the 20 random seeds.

**Figure 6:**
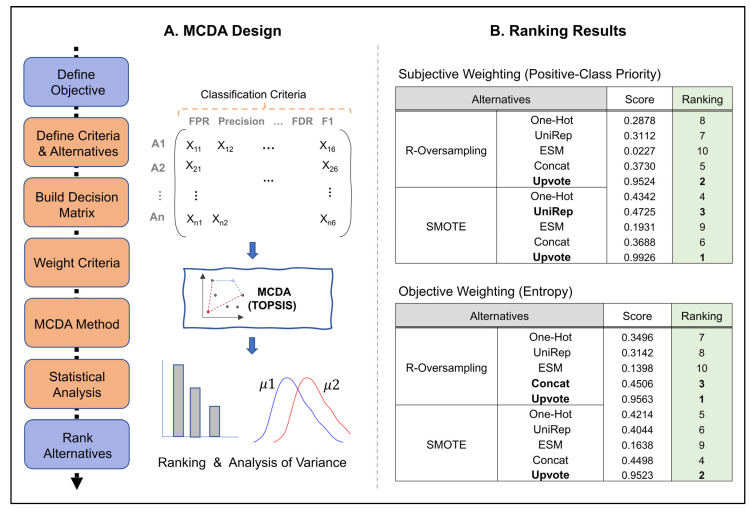
Upvoting achieved the best ranking both in subjective and objective weighting in MCDA design. A. The main steps for performing MCDA are elaborated. Then we highlighted our selected methods for implementing MCDA (e.g., classification criteria, model selection, and statistical analysis). B. TOPSIS scores (i.e., closeness coefficients) and their associated rankings are shown for subjective and objective weighting.

As shown in Figure 5 and Table S2, voting boosted the prediction score among the candidates, and SMOTE increased the performance in single encoders compared to R-oversampling. In order to obtain a more informed and transparent decision making among the mentioned methods, we also incorporated an MCDA with TOPSIS over five more classification metrics in addition to F1-score (refer to Section 2.7 for more details on the methods). These classification criteria were compared over single encoders, concat_all encoder, and upvoting technique. Figure 6 represents a summary of our MCDA design in addition to obtained ranking results from TOPSIS.

The MCDA design in the affibody dataset with only 7% high-fitness population enabled the comparison of encoding and sampling methods over multiple conflicting criteria. In addition, with selective weighting in MCDA, the user can bias the results toward more favorable results, based on data attributes and specific applications. Note that the rankings need to be validated by statistical analysis. Our MANOVA analysis showed significant results between the candidates. The Tukey method for pairwise comparison and family-wise correction error indicated that while upvoting methods achieved significant results over other candidates, there was no statistical difference between upvoting methods in using either SMOTE or R-oversampling. Table 2 is a summary reports of Tukey results among top candidates in rankings. A list of pair-wise comparisons over all alternatives can be found in the supplementary information.

**Figure 7:**
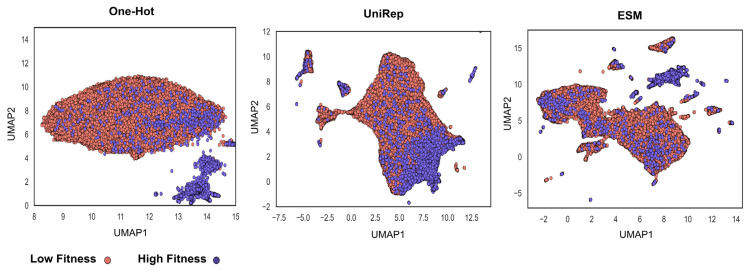
Different protein encodings potentially capture distinct functional aspects of the proteins. A 2D visualization of the encoding techniques that resulted in improved prediction in the voting method in UMAP. This method is a dimensionality reduction technique such as principal component analysis (PCA) [65] with unique advantages such as preserving the local structure of the data and capturing non-linear relationships between data points. In observing the sequence–function relationship in proteins, one can conclude that each protein sequence representation/encoding has the potential to capture different aspects of fitness.

The voting method enhanced the prediction score in both using a single metric and multiple metrics in MCDA by combining the predictions of multiple models based on single encodings. We concluded that as different encodings might capture the distance and relationship of the datapoints differently, combining their predictions boosted the final model performance. The encoding methods used for voting technique in the dataset are visualized in Figure 7 in a uniform manifold approximation and projection (UMAP) [64] plot.

### 3.5. How Protein Encodings Perform Considering Different Data Attributes

**Figure 8:**
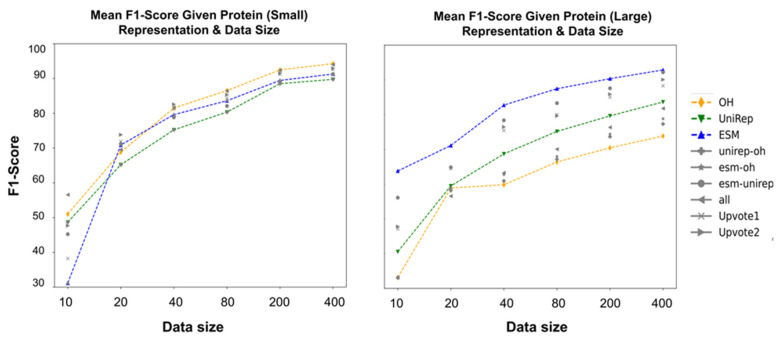
The effect of protein size on the performance of encoding methods in stability prediction while data sizes vary. The obtained results are largely different with respect to the protein size—small proteins (aa length ≤ 120) vs. large (400 ≤ aa length ≤ 1500). Highlights: For small proteins, upon comparing the violin plots and statistical test results, protein sequence encoding methods were performed distinctively with respect to the initial dataset (protein max length = 500). One-Hot encoding had a more significant contribution in boosting the classification metrics for small proteins. As an example, when *n* = 400, both One-Hot and All-Encoding concatenation with a mean F1-score of 94% outperformed the other encoding methods. One-Hot tends to be problematic for large proteins as it results in a highly sparse encoding vector. This was shown in this plot when One-Hot encoding performance was not satisfactory in comparison with ESM and UniRep. When *n* = 400, based on both the violin plots and the post-hoc analysis after ANOVA (both Bonferroni and Tukey), either ESM or ESM_UniRep with 92% mean F1-score achieved the highest performance. One-Hot with 73% mean F1-score was the lowest score among all the encodings. Refer to the supplementary information for all one-by-one comparisons of the statistics and classification.

The hypotheses were tested over affibody datasets that had notable attributes such as severe imbalance, multiple mutation sites, affinity and specificity enrichment, and small molecular protein length. The obtained results indicated voting and oversampling were highly effective methods to boost the fitness prediction performance. However, individual protein-encoding performance comparisons need more convincing explanation and thorough exploration. Specifically, we wondered why ESM underperformed One-Hot and UniRep despite more the powerful setup in pretraining and being showcased in studies for high prediction potential [66]. While the performance could be due to the datatype (e.g., small protein, complex fitness, etc.), we decided to further analyze the encoding prediction scores in a completely different dataset and bring insights on embedding performances in various conditions (e.g., data size in training, protein length, prediction task difficulty). The curated data contains 18,190 sequences with varying amino acid (aa) lengths and provides melting points that indicate the protein stability. Figure 8 is the performance comparison in the stability prediction of embeddings, their concatenation, and voting using different data sizes. Despite down performing in the affibody affinity data, ESM performed best for stability prediction when including proteins with max aa length = 500 (Figure S6).

We further evaluated the performance of embedding methods in large (400 ≤ aa length ≤ 1500) and small proteins (aa length ≤ 120) to check if ESM still outperforms the other representations in stability prediction. A complete list of statistical analysis is attached in the supplementary materials (Tables S1–S4). The analysis of physical feature encoding is provided in Figure S5.

The last analysis is a regression task for predicting the melting point value. We wondered how different encoding methods performed if we used all the data and increased the prediction challenge (Tm prediction rather than stability class prediction). MSE and R^2^ are shown predicting the Tm values of a dataset of 18,190 sequences with 0.3 test size. There was a significant difference in the performance of encoding methods, which was not the case in the classification task. ESM was the best encoding method in predicting stability (R^2^ score = 0.65). Note that we used all the data (i.e., did not use sampling) for training our regression model. The regression metrics are reported in Table 3.

## 4. Discussion

In this study, we shed light on two key challenges of applying discriminative models over amino acid sequence data for protein engineering applications: (1) handling imbalanced data and (2) choosing an appropriate protein representation (i.e., encoding). Assay-labeled sequence data in this domain is often severely imbalanced (due to the rugged and sparse nature of the protein fitness landscape) and requires careful consideration in data sampling, splitting, and choice in data representation for model training. To capture this common occurrence of imbalanced data, we trained discriminative ML models over our cytometry-sorted deep-sequenced small protein (affibody) data to distinguish between functional sequences (*n* = 6077) among a large collection of non-functional protein sequences (*n* = 82,663). We then explored the impact of encoding protein sequences using two simplistic approaches (One-Hot encoding, physiochemical encoding) and two language-based methods (UniRep, ESM). We hypothesized that as each protein representation may capture distinct information, combining representations via embedding concatenation and ensemble learning increases overall performance and generalizability.

To address the issue of imbalanced data, we implemented multiple sampling techniques—undersampling, random oversampling, and SMOTE—and compared performances via multiple classification metrics. Our results indicate that implementing oversampling techniques over imbalanced datasets improves predictive performance relative to undersampling or the exclusion of sampling methods. Among the sequence representation methods, embeddings are the answer to improved fitness prediction and data requirements. However, it is essential to consider the choice of protein representation, its benefits, and its drawbacks. For example, the choice of fitness to be predicted (e.g., thermal stability, binding affinity, target specificity) and the language model pretraining procedure affect the model’s predictive performance and need further discussion. Therefore, we analyzed an additional dataset (i.e., the NESP dataset, which included a variety of protein sequences with their Tm) to discuss the effect of protein representations over the variables such as protein length, protein fitness, and prediction type (i.e., classification vs. regression). For ensemble learning, we used majority voting to combine the prediction of each representation over the same ML model, which significantly improved the prediction score, and its obtained results were statistically significant using MANOVA and the post-hoc method (Figure 6, Table 2).

As only a very small fraction of protein sequences are experimentally annotated with properties, the primary goal of embeddings is to distill valuable information from unlabeled data and use them for property/fitness prediction. Previous reports have observed that there are sequence motifs, conserved regions, and evolutionary information in the protein databases that can be learned by language models [33,34,67]. This has been tested with different NLP techniques, varying model parameters, and clustering sizes for databases used and resulted in a wide array of language-based protein representations [30,66,68,69]. These promising embeddings (e.g., ESM, UniRep) have been evaluated in many studies and have improved the fitness prediction scores and alleviated the assay-labeled data requirements [37,68]. However, there are also studies that report minor improvements in predictions by using solely embedding methods. In some cases, prediction scores were improved by simpler representations such as One-Hot or physiochemical encoding [70,71]. Similarly, Rao et al. pointed out a different performance of embeddings in TAPE [34] with 38 million parameters based on different protein engineering tasks. Their model performed outstandingly in fluorescence and stability prediction while it did not perform as well as hand-engineered features in contact prediction.

The current capabilities and limitations of language models motivate the need for optimizing the pretraining task and improving the methodology for supervising the pre-trained models. Consider ESM2, one of the largest language models used for protein sequences that has shown significant improvement in protein structure prediction compared to previous models. In our study, protein representations obtained via ESM2 significantly outperformed UniRep or One-Hot in stability prediction. However, in the context of predicting binding functionality among small protein affibody variants, its performance was exceeded by UniRep and One-Hot (Figure 4). This motivates looking into what knowledge is transferred by pretraining models and how useful they are for specific fitness predictions, with or without further supervision. Here, we covered the core challenges and considerations in supervising the models in fitness prediction, yet additional downstream analysis and posing insightful questions will give us more understanding and directions in discriminating the protein sequences based on their fitness. In order to improve the pretraining step, we might adopt techniques such as adjusting the masking rate [72], adding biological priors [69,73], increasing the model parameters [66], and building specialized language models for the desired fitness [74], given the growing data availability and computational resources. Additional studies are required for improved downstream fitness predictions, such as fine-tuning with a reduced chance of overfitting [75], incorporating the effect of post-translational modifications, and characterizing the performance of embeddings in different data setups [76] with varying protein types and finesses for supporting the development of novel proteins in diagnostics and therapeutics.

## 5. Conclusions

This study intends to inform protein engineers that: (i) embeddings derived from self-supervised representation techniques are not always the optimal route to take, depending on the protein size and protein fitness to be predicted; (ii) oversampling techniques, especially SMOTE, have the ability to overcome the notorious challenge of highly imbalanced data in the protein fitness landscape; (iii) different aspects learned in each protein encoding can be combined by voting techniques and result in better predictive scores. These conclusions were revealed in the context of integrating machine learning and protein engineering knowledge to identify high-fitness protein sequences. Specifically, we quantified model performance while varying the choice of feature representation, ensemble learning, and sampling methods. Analysis across a broad range of protein chain lengths revealed the ESM language model to be most beneficial for encoding large protein sequences (Figure 8). However, in the context of small protein sequences, a comparable performance was observed between One-Hot encoding and the language models (ESM and UniRep). In our analysis, oversampling proved to be an effective technique to improve performance when dealing with severely imbalanced datasets (Figure 4). Finally, ensemble learning was a promising method for boosting the binding prediction scores when using unique, competitive encoding methods (Figure 5 and Figure 6).

## Figures and Tables

**Table 2:** Tukey results over all classification metrics between selected representation methods.

Comparison	Mean GP1	Mean GP2	Metrics	Reject Null
	0.9712	0.9682	F1	FALSE
Upvote_SM	0.0187	0.0258	FDR	FALSE
vs.	0.9614	0.9622	TPR	FALSE
Upvote_RO	0.9813	0.9742	Precision	FALSE
	0.9972	0.9972	NPV	FALSE
	0.0019	0.0013	FPR	FALSE
	0.9712	0.9307	F1	TRUE
Upvote_SM	0.0187	0.0930	FDR	TRUE
vs.	0.9614	0.9557	TPR	TRUE
UniRep_SM	0.9813	0.9070	Precision	TRUE
	0.9972	0.9967	NPV	TRUE
	0.0019	0.0072	FPR	TRUE
	0.9712	0.9261	F1	TRUE
Upvote_SM	0.0187	0.1061	FDR	TRUE
vs.	0.9614	0.9607	TPR	FALSE
Concat_RO	0.9813	0.8939	Precision	TRUE
	0.9972	0.9971	NPV	FALSE
	0.0019	0.0084	FPR	TRUE
	0.9682	0.9307	F1	TRUE
Upvote_RO	0.0258	0.0930	FDR	TRUE
vs.	0.9622	0.9557	TPR	TRUE
UniRep_SM	0.9742	0.9070	Precision	TRUE
	0.9972	0.9967	NPV	TRUE
	0.0013	0.0072	FPR	TRUE
	0.9682	0.9261	F1	TRUE
Upvote_RO	0.0258	0.1061	FDR	TRUE
vs.	0.9622	0.9607	TPR	FALSE
Concat_RO	0.9742	0.8939	Precision	TRUE
	0.9972	0.9971	NPV	FALSE
	0.0013	0.0084	FPR	TRUE
	0.9261	0.9307	F1	TRUE
Concat_RO	0.1061	0.0930	FDR	TRUE
vs.	0.9607	0.9557	TPR	TRUE
UniRep_SM	0.8939	0.9070	Precision	TRUE
	0.9971	0.9967	NPV	TRUE
	0.0084	0.0072	FPR	TRUE

**Table 3:** Regression metrics for encoding methods in validation and test.

Encoding	Validation		Test	
	R²	MSE	R²	MSE
One-Hot	0.21	141	0.24	130
UniRep	0.49	108	0.40	102
ESM	0.65	63	0.65	60

## Data Availability

The NESP data are available at https://www.kaggle.com/competitions/novozymes-enzyme-stability-prediction/data (accessed on 25 October 2022). The Affibody dataset is available upon request the source code for this project can be found on the GitHub repository: https://github.com/WoldringLabMSU/Sequence_Fitness_Prediction.

## Supplementary Materials

The following supporting information can be downloaded at: https://www.mdpi.com/article/10.3390/pharmaceutics15051337/s1, Figure S1: Physicochemical feature correlation plot for affibody dataset. Figure S2: Confusion matrix results for the affibody dataset given single encodings, encoding con-catenation, and upvoting. Figure S3: Physicochemical feature correlation plot NESP dataset. Figure S4: Physicochemical feature ranking for NESP dataset. Figure S5: Physical feature representation while using maximum *n* = 1000 performed poorly and was not selected for the main figure. Figure S6: Mean F1-score comparison between protein representations including proteins with max length = 500. Figure S7: A complete list of used criteria for MCDA and their derivations from confusion matrix values. Table S1: Figure 4 Statistical analysis for analyzing significance of the results and ranking the methods based on their performance. This analysis is performed with initial one-way ANOVA accompanied by Bonferroni post hoc to account for family-wise error rate and rank the methods based on their performance. Table S2: Figure 5 Statistical analysis for analyzing significance of the results and ranking the methods based on their performance. This analysis is performed with initial one-way ANOVA accompanied by Bonferroni post hoc to account for family-wise error rate and rank the methods based on their performance. Table S3: Statistical analysis for R-Oversampling vs. SMOTE. SMOTE performed significantly better than oversampling in ESM, One-Hot, and UniRep encodings while its performance was similar to R-Oversampling in techniques such as upvoting and physical feature encoding. Table S4: Statistical analysis for Figure S2. Note that Tukey method results are provided in csv files.
