# Supplementary material for: Protein Fitness Prediction Is Impacted by the Interplay of Language Models, Ensemble Learning, and Sampling Methods
Source: Pharmaceutics. 2023 Apr 25;15(5):1337. doi: 10.3390/pharmaceutics15051337 (PMC10224321; doi:10.3390/pharmaceutics15051337)
Supplement: Supplementary file 1 [file pharmaceutics-15-01337-s001.zip › Supplement_complete.pdf]

Supplemental Information

Protein Fitness Prediction is Impacted by the Interplay of Language Models, Ensemble Learning, and Sampling Methods

Mehrsa Mardikoraem, Daniel Woldring

Introduction

In the manuscript, two datasets (affibody and NESP) with different fitness attributes (stability and binding affinity) were selected to not only compare the protein representations and sampling methods but also to explain how they perform in important fitness prediction tasks. As an example, language models, such as ESM, have been trained over millions of protein sequences in public databases and have produced high performance in certain fitness tasks (e.g., stability prediction) while they may not do as well in all fitness tasks. This paper aims to emphasize the extra care that users should consider when using language model techniques based on their intended application. In addition, we explored several methods to increase the model's predictive performance (e.g., using sampling methods and voting techniques). While many possible factors (e.g., encoding and sampling methods, performance metrics used) may influence the results, we limited our search space to focus on four well-represented and highly used encodings ((One-hot, Physiochemical Encoding, UniRep[32], and ESM[33]), as well as three sampling methods which are relevant to highly imbalanced datasets.

Protein Fitness: the ability of a protein to perform its biological function or functions effectively and efficiently within a specific environment or context. A protein's fitness is influenced by a variety of factors, including its structure, stability, and interactions with other molecules. Proteins with high fitness are able to carry out their intended roles with minimal errors or deviations from their normal function, whereas proteins with low fitness may have reduced activity, altered specificity, or decreased stability.

Statistical Analysis: For pair-wise comparisons, we performed Student's t-test for identifying whether any potential differences between methods are significant. Moreover, to account for multiple comparisons among all groups, we added an analysis of variance (ANOVA; MANOVA when multiple metrics are included) test to first identify if there were any significant results among these groups. Performing ANOVA between our candidates for encoding and sampling methods, the obtained result showed that the results are significant. Then, we conducted post hoc analyses to account for the family-wise error rates caused by multiple comparisons to reduce the type-1 error rate. Implementing ANOVA followed by multiple post hoc statistical methods (Bonferroni and Tukey), we were able to rank their performances. We have implemented both the Bonferroni correction and Tukey method for the post hoc analysis and made the results available in the supplementary information (Table S1-S3). Both methods were largely in agreement on rejecting/accepting the null hypothesis (i.e., no significant difference between group i vs group j). The complete set of comparisons for both post hoc statistical methods can be found in the attached CSV in the supplementary information. Yet, due to the considerable quantity of analyses conducted (e.g. 120 combinatorial comparisons for representations and sampling methods), only the statistics directly associated with main manuscript figures are included here in the supplement.

Selection of ML parameters: We have taken several steps to ensure a fair comparison between methods. To account for a fair comparison and adequately support our conclusions, we have compiled extensive performance metrics within supplementary information (Figure S1-S6; Table S1-S4). Specifically, we would like to justify our choices in terms of ML model selection, performance metric selection, and encoding choice. For ML model selection, we opted for simple logistic regression for classification as it is less sensitive to parameter selection and therefore appropriate for comparing methods[1]. For regression analysis, we conducted hyperparameter optimization based on each encoding method to ensure the model parameters were optimized based on the encoding input attributes. Regarding performance metric selection, we acknowledge that there are numerous criteria available for evaluating an ML model. However, given the unique protein fitness landscape attributes (we mainly focus on identifying the rare positive instances among a pool consisting of mostly low-fitness sequences), we initiated our decision-making process using the F1 score for classification and R-squared and MSE for regression as reliable measures for evaluating model performance. Then, to incorporate a more complete assessment of our encoding and sampling approaches, we included a total of six criteria: F1-score, precision, FPR, TPR, FDR, and NPV. To assess model performance in the context of multiple conflicting criteria, we implemented multiple criteria decision analysis (MCDA) with TOPSIS. MCDA enables ranking alternatives while taking into account each performance criterion.

In this way, among virtually endless factors, we reduced our search space by carefully choosing fair ML models for comparisons, practical and well-known encodings, and specialized performance metrics based on the protein fitness landscape.

While our study's results hold true in the context of these protein attributes, generalizing these results to encompass all proteins will require more extensive studies. Yet, we aimed to incorporate various aspects of the protein fitness landscape in our study (e.g., different protein fitness and different protein sizes). Note that while we believe our results could derive generalizable interpretations, this is indisputable that different data sets might result in fairly different interpretations. However, this study intends to inform protein engineers that:

i) Language model encoders are not always the definite route to take depending on the protein size and protein fitness to be predicted.
ii) Oversampling techniques, especially SMOTE, has the ability to overcome the notorious challenge of highly imbalanced data in the protein fitness landscape.
iii) Different aspects learned in each protein encoding can be combined by voting techniques and result in better predictive scores.

Correlation Plot for Physical Features, Affibody Dataset Sample

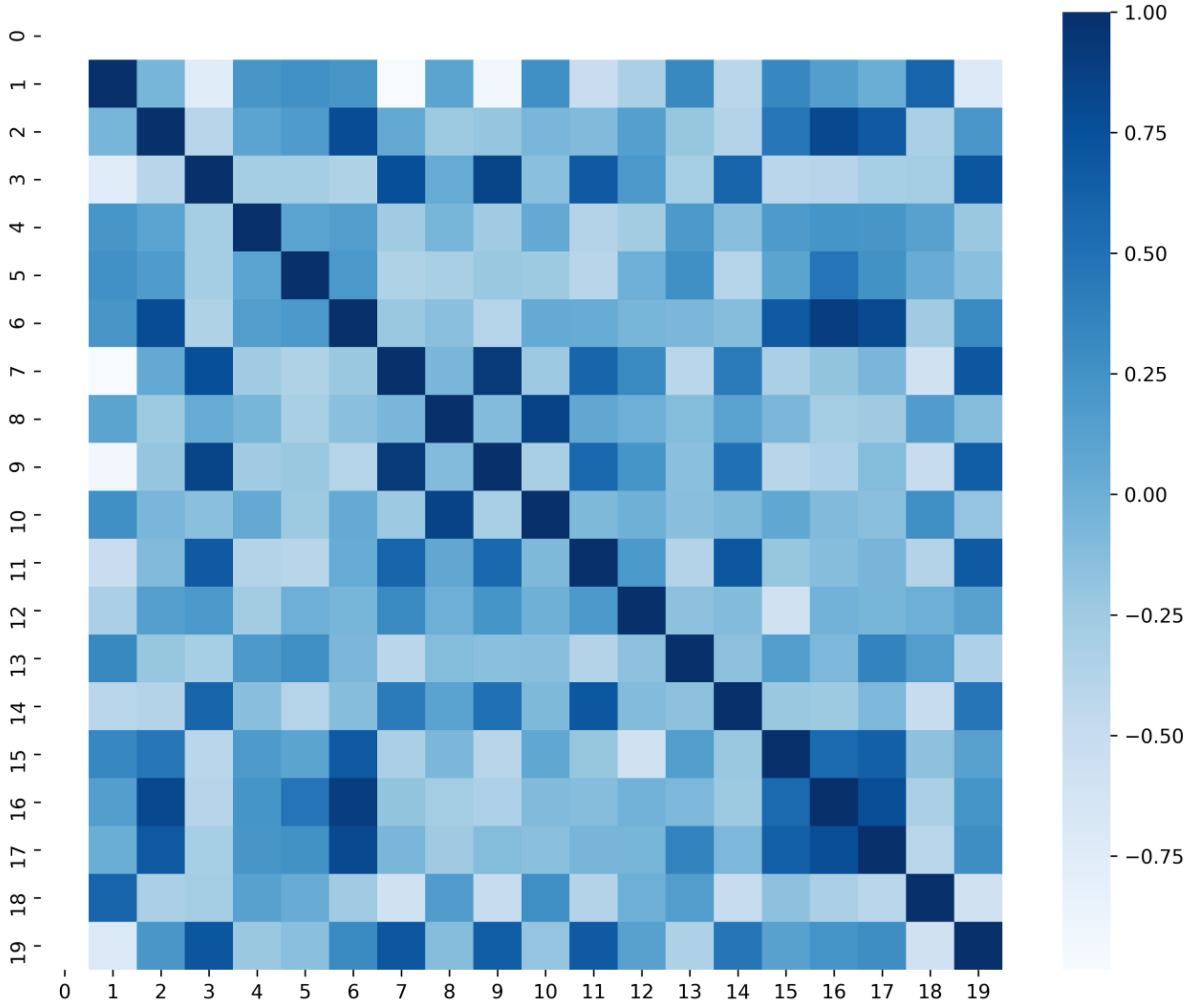

Figure S1. The majority of physical features are at least correlated with one other physical feature; leaving us insights into attribute dependencies in the affibody dataset. The plot is drawn upon sampling from both naïve and enriched populations. Bold colors represent a higher correlation of features. As the affibody length in our dataset was fixed, the first row and column of the matrix are empty. Insights on the given correlation plot include i) a high correlation of Boman index and protein flexibility index, ii) a high correlation of aromaticity with molecular weight, MSW, and refractivity, iii) Aliphatic index having a correlation with multiple features such as bulkiness, H_Eisenberg, and H_Gravy.

0)L, 1)Boman,2)Aromaticity, 3)Aliphatic,4)Instability, 5)Charge, 6)MW, 7)H_Eisenberg, 8)uH_Eisenberg, 9H)_GRAVY,10)uH_GRAVY, 11)Z3_1, 12)Z3_2, 13)Z3_3,14)Levitt alpha, 15)MSS, 16)MSW, 17)refractivity, 18)flexibility, 19)bulkiness

Table S1: Figure 4 T-test Results

	Comparison	P-Value
Under-sampling	Physical vs. **OH**	**2.37E-27**
	Physical vs. **UniRep**	**2.60E-24**
	Physical vs. **ESM**	**2.22E-20**
	OH vs. **UniRep**	**6.86E-06**
	**OH** vs. ESM	**9.31E-13**
	**UniRep** vs. ESM	**6.44E-08**
R-oversampling	Physical vs. **OH**	**6.12E-52**
	Physical vs**. UniRep**	**3.20E-50**
	Physical vs. **ESM**	**5.11E-50**
	OH vs. UniRep	0.619
	**OH** vs. ESM	**1.06E-18**
	**UniRep** vs. ESM	**9.01E-17**
SMOTE	Physical vs. **OH**	**2.06E-51**
	Physical vs. **UniRep**	**2.87E-50**
	Physical vs. **ESM**	**1.15E-50**
	OH vs. UniRep	0.223
	**OH** vs. ESM	**9.29E-15**
	**UniRep** vs. ESM	**9.01E-17**

The initial ANOVA test was done for multiple comparisons, and it resulted in p-value= 9.53E-190.

The following tables in the supplement show the t-test results when Bonferroni correction is considered. The statistically significant results are bolded.

Please refer to csv files for additional post-hoc analysis.

Table S2: Figure 5 T-test Results-part1

Comparisons R-Oversampling for i vs. j	Mean i	Mean j	P-value
OH-vs-UniRep	91.92	92	0.619375
OH-vs-ESM	91.92	89.91	**1.06E-18**
OH-vs-UniRep+OH	91.92	92.54	**0.000201**
OH-vs-ESM+OH	91.92	91.45	0.026281
OH-vs-ESM+UniRep	91.92	90.56	**1.77E-09**
OH-vs-All	91.92	92.61	**8.38E-05**
OH-vs-Upvoted	91.92	96.81	**2.97E-31**
UniRep-vs-ESM	92	89.91	**2.04E-16**
UniRep-vs-UniRep+OH	92	92.54	0.002124
UniRep-vs-ESM+OH	92	91.45	0.015463
UniRep-vs-ESM+UniRep	92	90.56	**1.57E-09**
UniRep-vs-All	92	92.61	**0.000896**
UniRep-vs-Upvoted	92	96.81	**8.12E-26**
ESM-vs-UniRep+OH	89.91	92.54	**4.29E-19**
ESM-vs-ESM+OH	89.91	91.45	**2.15E-08**
ESM-vs-ESM+UniRep	89.91	90.56	**0.000344**
ESM-vs-All	89.91	92.61	**9.44E-19**
ESM-vs-Upvoted	89.91	96.81	**2.52E-38**
UniRep+OH-vs-ESM+OH	92.54	91.45	**1.47E-05**
UniRep+OH-vs-ESM+UniRep	92.54	90.56	**4.37E-13**
UniRep+OH-vs-All	92.54	92.61	0.706828
UniRep+OH-vs-Upvoted	92.54	96.81	**8.42E-24**
ESM+OH-vs-ESM+UniRep	91.45	90.56	**0.000355**
ESM+OH-vs-All	91.45	92.61	**7.00E-06**
ESM+OH-vs-Upvoted	91.45	96.81	**1.35E-19**
ESM+UniRep-vs-All	90.56	92.61	**2.59E-13**
ESM+UniRep-vs-Upvoted	90.56	96.81	**2.84E-25**
All-vs-Upvoted	92.61	96.81	**8.43E-23**

Bonferroni Correction for Rejecting Null Hypothesis

$$\alpha = 0.05/120 \cong 0.0004$$

Table S2: Figure 5 T-test Results-part 2

Comparisons for SMOTE for i vs. j	Mean i	Mean j	P-value
OH-vs-UniRep	92.88	93.07	0.222968
OH-vs-ESM	92.88	91.09	**9.29E-15**
OH-vs-UniRep+OH	92.88	92.53	0.050908
OH-vs-ESM+OH	92.88	91.61	**5.80E-11**
OH-vs-ESM+UniRep	92.88	90.47	**1.85E-15**
OH-vs-All	92.88	92.58	0.074292
OH-vs-Upvoted	92.88	97.08	**4.02E-24**
UniRep-vs-ESM	93.07	91.09	**9.01E-17**
UniRep-vs-UniRep+OH	93.07	92.53	0.003022
UniRep-vs-ESM+OH	93.07	91.61	**3.23E-13**
UniRep-vs-ESM+UniRep	93.07	90.47	**1.72E-16**
UniRep-vs-All	93.07	92.58	0.004057
UniRep-vs-Upvoted	93.07	97.08	**6.46E-25**
ESM-vs-UniRep+OH	91.09	92.53	**2.43E-10**
ESM-vs-ESM+OH	91.09	91.61	**0.000153**
ESM-vs-ESM+UniRep	91.09	90.47	0.000995
ESM-vs-All	91.09	92.58	**1.50E-11**
ESM-vs-Upvoted	91.09	97.08	**1.88E-32**
UniRep+OH-vs-ESM+OH	92.53	91.61	**1.56E-06**
UniRep+OH-vs-ESM+UniRep	92.53	90.47	**8.55E-13**
UniRep+OH-vs-All	92.53	92.58	0.792126
UniRep+OH-vs-Upvoted	92.53	97.08	**1.11E-21**
ESM+OH-vs-ESM+UniRep	91.61	90.47	**1.11E-07**
ESM+OH-vs-All	91.61	92.58	**1.50E-07**
ESM+OH-vs-Upvoted	91.61	97.08	**2.07E-34**
ESM+UniRep-vs-All	90.47	92.58	**1.74E-13**
ESM+UniRep-vs-Upvoted	90.47	97.08	**1.37E-24**
All-vs-Upvoted	92.58	97.08	**4.06E-23**

Bonferroni Correction for Rejecting Null Hypothesis

$$\alpha = 0.05/120 \cong 0.0004$$

Table S3: SMOTE either Improved the performance or had no hampering effect with respect to R-Oversampling**.**

**Comparison for Samplings R-Oversampling vs. SMOTE**	**Mean R-Oversampling**	**Mean SMOTE**	**P-Value**
OH	91.92	92.88	**7.30e-08**
UniRep	92	93.07	**3.08e-08**
ESM	89.91	91.09	**1.90e-11**
UniRep+OH	92.54	92.53	0.96
ESM+OH	91.45	91.61	0.44
ESM+UniRep	90.56	90.47	0.65
All	92.61	92.58	0.88
Upvoted	96.81	97.08	0.002

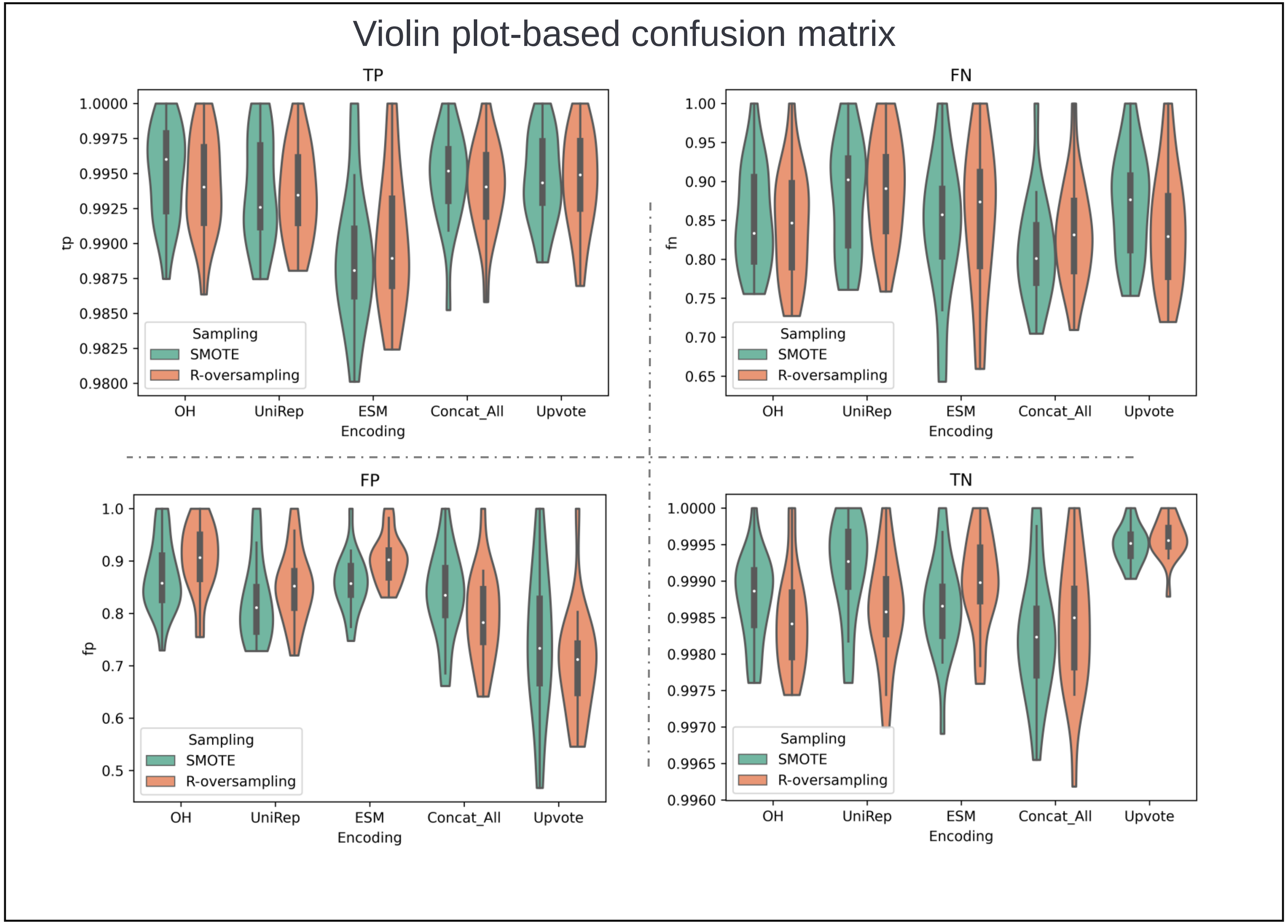

Figure S2. **Individual Encodings Perform uniquely in the confusion matrix entities.** While the overall predictive performance is the main goal and it is represented via F1-Score throughout the literature, inspecting how each model performs for maximizing true positives(TP) and true negatives(TN) while minimizing false positives(FP) and false negatives(FN), provides insights about each model performance.

Table S4. T-Test for Figure S4

Stat for TP

Encoding	P-value	Mean i	Mean j	Sampling
OH-vs-UniRep	0.532	0.994	0.993	R-Oversampling
OH-vs-ESM	0.005	0.994	0.990	R-Oversampling
OH-vs-Concat_All	0.861	0.994	0.994	R-Oversampling
OH-vs-Upvote	0.848	0.994	0.994	R-Oversampling
UniRep-vs-ESM	0.016	0.993	0.990	R-Oversampling
UniRep-vs-Concat_All	0.634	0.993	0.994	R-Oversampling
UniRep-vs-Upvote	0.410	0.993	0.994	R-Oversampling
ESM-vs-Concat_All	0.006	0.990	0.994	R-Oversampling
ESM-vs-Upvote	0.003	0.990	0.994	R-Oversampling
Concat_All-vs-Upvote	0.707	0.994	0.994	R-Oversampling
OH-vs-UniRep	0.340	0.995	0.994	SMOTE
OH-vs-ESM	**1.03E-4**	0.995	0.989	SMOTE
OH-vs-Concat_All	0.764	0.995	0.995	SMOTE
OH-vs-Upvote	0.760	0.995	0.995	SMOTE
UniRep-vs-ESM	**0.002**	0.994	0.989	SMOTE
UniRep-vs-Concat_All	0.493	0.994	0.995	SMOTE
UniRep-vs-Upvote	0.480	0.994	0.995	SMOTE
ESM-vs-Concat_All	**1.95E-4**	0.989	0.995	SMOTE
ESM-vs-Upvote	**1.65E-4**	0.989	0.995	SMOTE
Concat_All-vs-Upvote	0.998	0.995	0.995	SMOTE

Stat for FN

Encoding	P-value	Mean i	Mean j	Sampling
OH-vs-UniRep	0.041	0.844	0.890	R-Oversampling
OH-vs-ESM	0.77	0.844	0.852	R-Oversampling
OH-vs-Concat_All	0.609	0.844	0.833	R-Oversampling
OH-vs-Upvote	0.88	0.844	0.840	R-Oversampling
UniRep-vs-ESM	0.152	0.890	0.852	R-Oversampling
UniRep-vs-Concat_All	0.01	0.890	0.833	R-Oversampling
UniRep-vs-Upvote	0.034	0.890	0.840	R-Oversampling
ESM-vs-Concat_All	0.47	0.852	0.833	R-Oversampling
ESM-vs-Upvote	0.679	0.852	0.840	R-Oversampling
Concat_All-vs-Upvote	0.734	0.833	0.840	R-Oversampling
OH-vs-UniRep	0.281	0.854	0.878	SMOTE
OH-vs-ESM	0.59	0.854	0.840	SMOTE
OH-vs-Concat_All	0.054	0.854	0.812	SMOTE
OH-vs-Upvote	0.463	0.854	0.870	SMOTE
UniRep-vs-ESM	0.153	0.878	0.840	SMOTE
UniRep-vs-Concat_All	0.005	0.878	0.812	SMOTE
UniRep-vs-Upvote	0.708	0.878	0.870	SMOTE
ESM-vs-Concat_All	0.265	0.840	0.812	SMOTE
ESM-vs-Upvote	0.251	0.840	0.870	SMOTE
Concat_All-vs-Upvote	0.01	0.812	0.870	SMOTE

Bonferroni Correction for Rejecting Null Hypothesis $\alpha = \frac{0.05}{45} \cong 0.001$

Table S4. T-Test for Figure S4

Stat for TN

Encoding	P-value	Mean i	Mean j	Sampling
OH-vs-UniRep	0.569	0.9984	0.9986	R-Oversampling
OH-vs-ESM	0.018	0.9984	0.9990	R-Oversampling
OH-vs-Concat_All	0.758	0.9984	0.9984	R-Oversampling
OH-vs-Upvote	**4.60E-07**	0.9984	0.9996	R-Oversampling
UniRep-vs-ESM	0.081	0.9986	0.9990	R-Oversampling
UniRep-vs-Concat_All	0.429	0.9986	0.9984	R-Oversampling
UniRep-vs-Upvote	**7.12E-6**	0.9986	0.9996	R-Oversampling
ESM-vs-Concat_All	0.021	0.9990	0.9984	R-Oversampling
ESM-vs-Upvote	**0.001**	0.9990	0.9996	R-Oversampling
Concat_All-vs-Upvote	**9.80E-06**	0.9984	0.9996	R-Oversampling
OH-vs-UniRep	0.340	0.9987	0.9992	SMOTE
OH-vs-ESM	**1.03E-4**	0.9987	0.9986	SMOTE
OH-vs-Concat_All	0.764	0.9987	0.9982	SMOTE
OH-vs-Upvote	0.760	0.9987	0.9995	SMOTE
UniRep-vs-ESM	0.002	0.9992	0.9986	SMOTE
UniRep-vs-Concat_All	0.493	0.9992	0.9982	SMOTE
UniRep-vs-Upvote	0.480	0.9992	0.9995	SMOTE
ESM-vs-Concat_All	**1.95E-4**	0.9986	0.9982	SMOTE
ESM-vs-Upvote	**1.65E-4**	0.9986	0.9995	SMOTE
Concat_All-vs-Upvote	0.998	0.9982	0.9995	SMOTE

Stat for FP

Encoding	P-value	Mean i	Mean j	Sampling
OH-vs-UniRep	0.022	0.903	0.852	R-Oversampling
OH-vs-ESM	0.975	0.903	0.903	R-Oversampling
OH-vs-Concat_All	**7.37E-05**	0.903	0.794	R-Oversampling
OH-vs-Upvote	**2.54E-08**	0.903	0.703	R-Oversampling
UniRep-vs-ESM	0.01	0.852	0.903	R-Oversampling
UniRep-vs-Concat_All	0.025	0.852	0.794	R-Oversampling
UniRep-vs-Upvote	**6.07E-06**	0.852	0.703	R-Oversampling
ESM-vs-Concat_All	**2.71E-05**	0.903	0.794	R-Oversampling
ESM-vs-Upvote	**2.10E-08**	0.903	0.703	R-Oversampling
Concat_All-vs-Upvote	0.05	0.794	0.703	R-Oversampling
OH-vs-UniRep	0.501	0.872	0.824	SMOTE
OH-vs-ESM	0.131	0.872	0.858	SMOTE
OH-vs-Concat_All	0.001	0.872	0.834	SMOTE
OH-vs-Upvote	0.121	0.872	0.743	SMOTE
UniRep-vs-ESM	0.692	0.824	0.858	SMOTE
UniRep-vs-Concat_All	0.033	0.824	0.834	SMOTE
UniRep-vs-Upvote	0.291	0.824	0.743	SMOTE
ESM-vs-Concat_All	0.002	0.858	0.834	SMOTE
ESM-vs-Upvote	0.018	0.858	0.743	SMOTE
**Concat_All-vs-Upvote**	0.05	0.834	0.743	SMOTE

Bonferroni Correction for Rejecting Null Hypothesis $\alpha = \frac{0.05}{45} \cong 0.001$

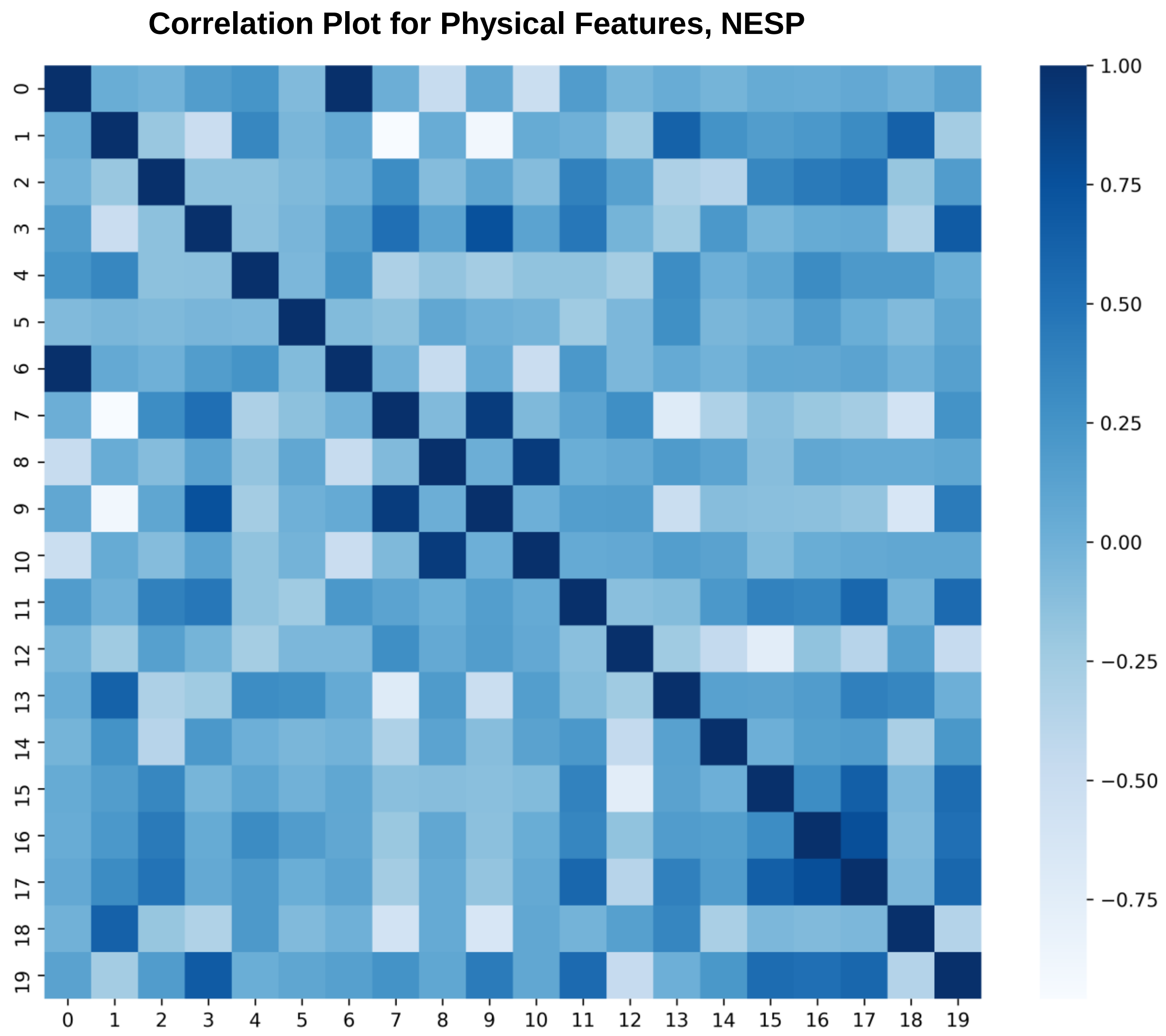

Note: From this Figure (Figure S2) the results are generated from NESP data**.**

0)L, 1)Boman,2)Aromaticity, 3)Aliphatic,4)Instability, 5)Charge, 6)MW, 7)H_Eisenberg, 8)uH_Eisenberg, 9H)_GRAVY,10)uH_GRAVY, 11)Z3_1, 12)Z3_2, 13)Z3_3,14)levitt_alpha, 15)MSS, 16)MSW, 17)refractivity, 18)flexibility, 19)bulkiness

Figure S3. Physical feature correlation plot for NESP dataset.

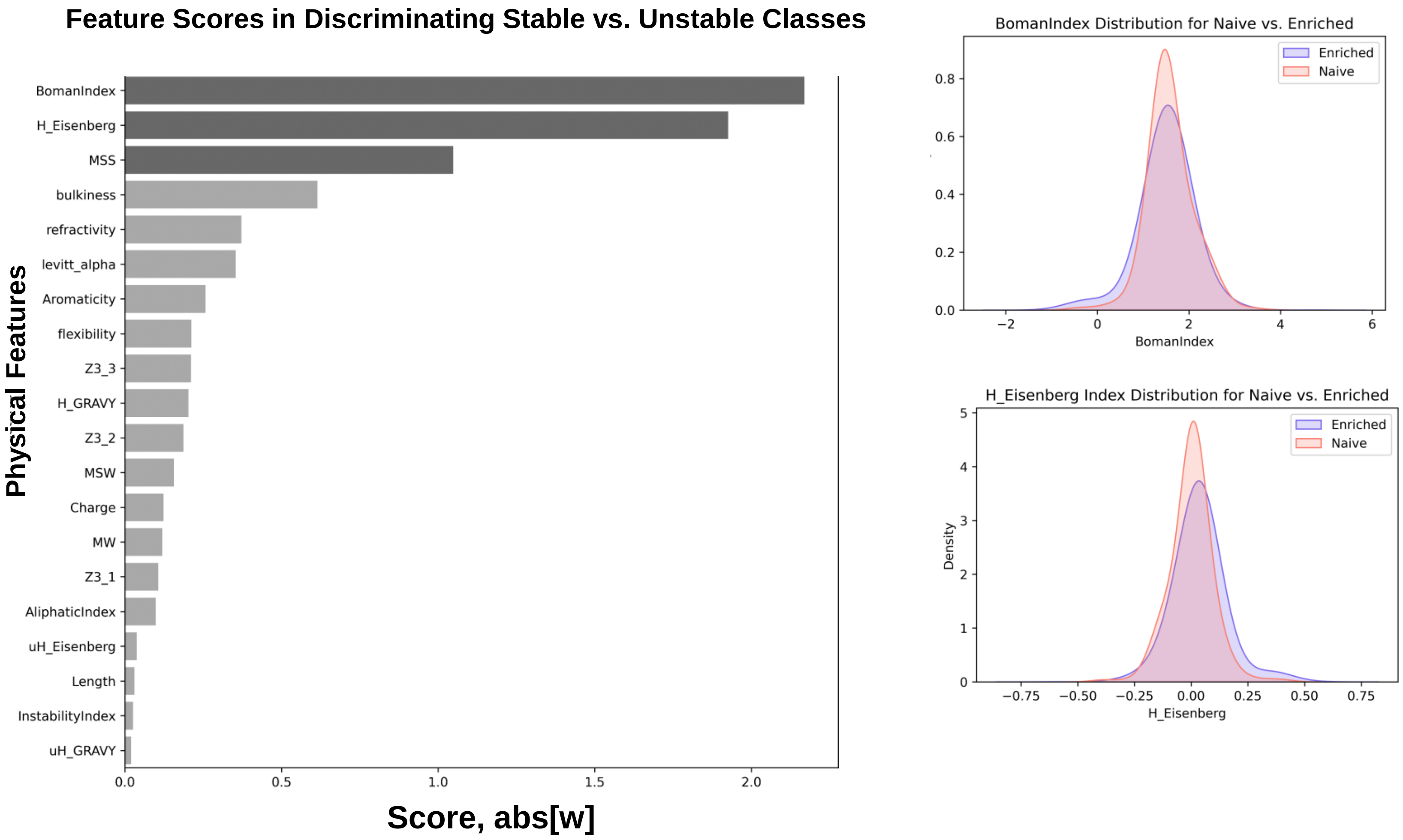

Figure S4: Physical feature ranking for NESP dataset. The feature ranking is presented after using all the data for the classification of stable vs. unstable sequences. Boman Index, H_Eisenberg, and MSS are the lead features. However, their scores are not significantly higher than the other physical attributes, indicating that more features incorporate in the final F1_Score=0.86.

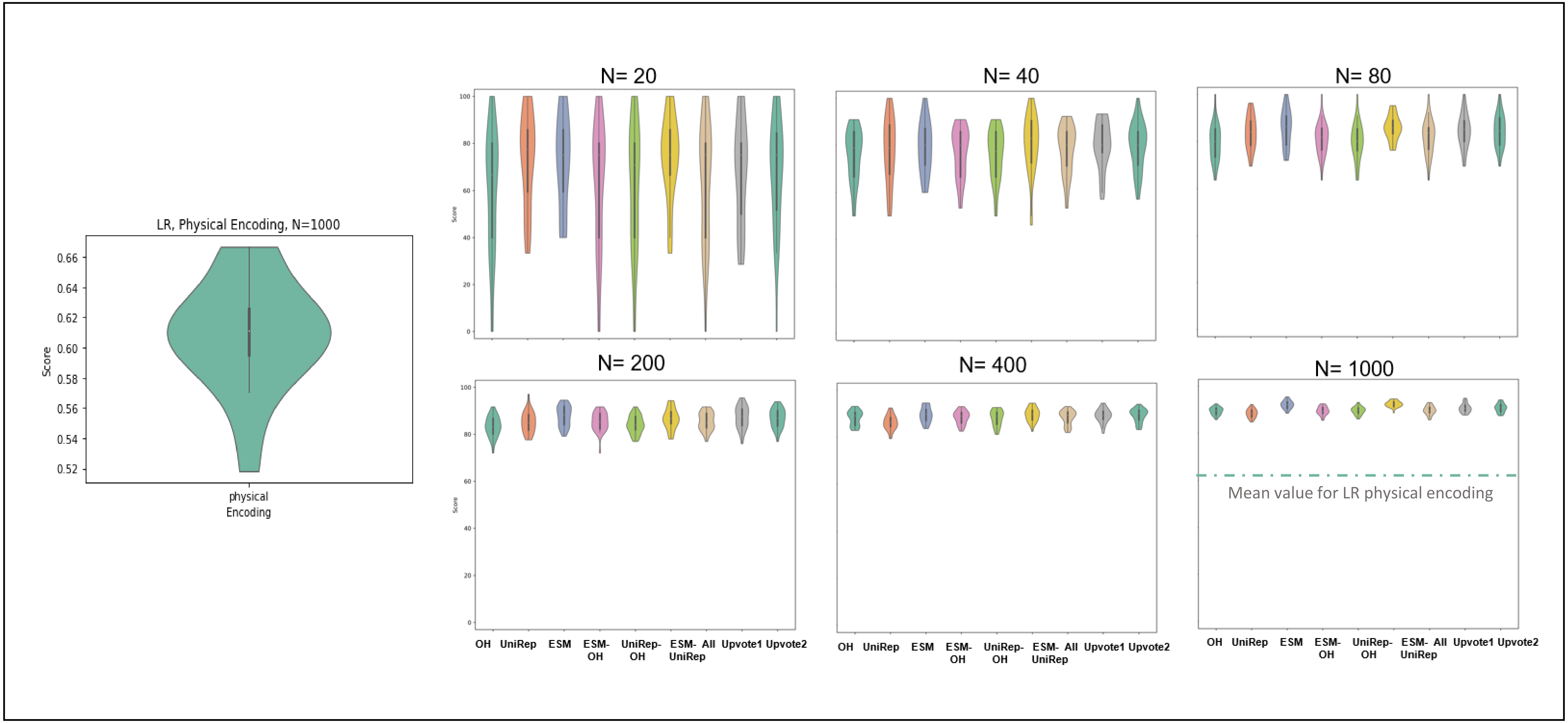

Figure S5. Physical Feature representation while using maximum N=1000, performed poorly and have not got selected for the main figure.

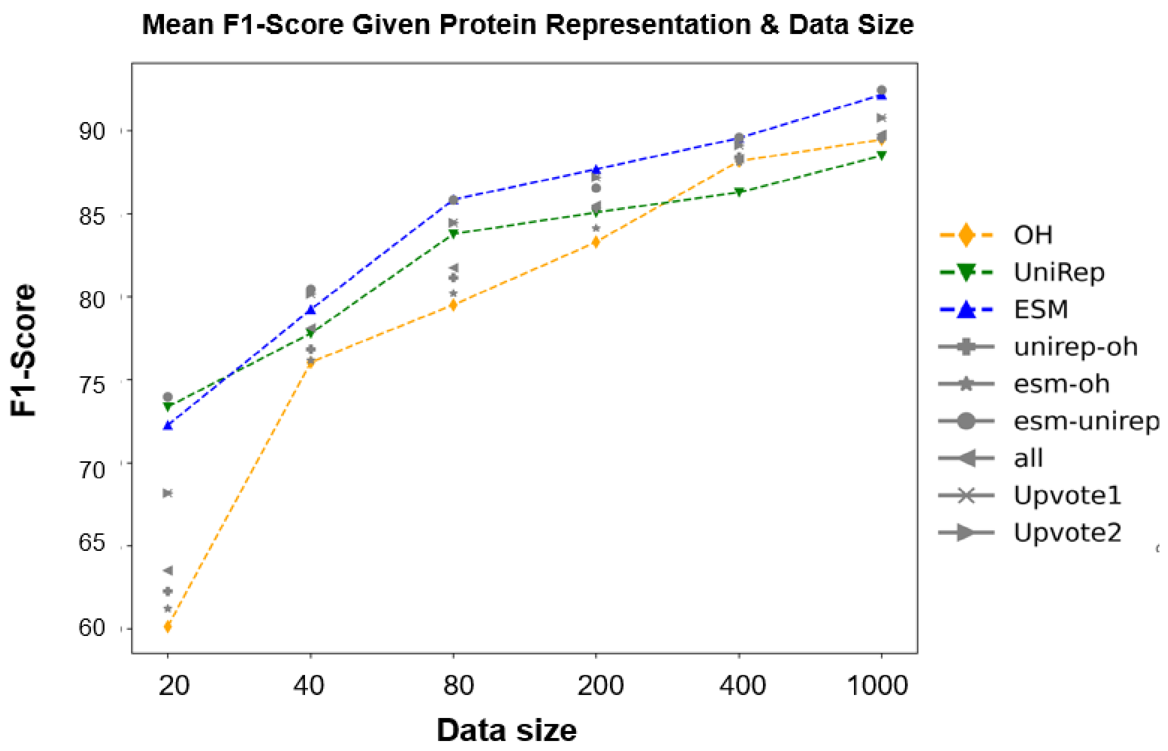

Figure S6. The predictive performances (F1-score) of multiple protein representations (One-Hot, UniRep, and ESM) were evaluated across stable (Tm>=60C; n=3140) vs. unstable (Tm<= 35C; n=1116) proteins. The performance of individual representations is compared against the effects of concatenating each embedding as well as ensemble methods (upvote1: hard voting, upvote2: soft voting). The violin plots were generated by repeating the analysis over 30 random seeds for sensitivity analysis. N represents the total number of data used with 0.3 as a test-size ratio. Welch t-test with unequal variances has been implemented over the obtained results to showcase the statistical significance in comparisons (refer to supplementary information for p-values). Highlight: In low data size (N=20), One-Hot performs poorly with the mean F1-score of 0.60, and the embeddings that included One-Hot were outperformed by both ESM and UniRep. While increasing the data size resulted in increased performance for all the methods, concatenating ESM with UniRep representations obtained the best score, with a mean F1 of 0.92.

Metric	Equation
F_1	$\frac{2 * Precision * Recall}{Precision + Recall}$
Precision	$\frac{TP}{TP + FP}$
Recall, TPR	$\frac{TP}{TP + FN}$
FPR	$\frac{FP}{FP + TN}$
FDR	$\frac{FP}{FP + TP}$
NPV	$\frac{TN}{TN + FN}$

Figure S7: A complete list of used criteria for MCDA & their derivations from confusion matrix values
